# Pre-pregnancy LDL/HDL and total Cholesterol/HDL ratios are strong predictors of gestational diabetes mellitus in women undergoing assisted reproductive technologies

**DOI:** 10.1186/s12958-024-01320-9

**Published:** 2024-12-05

**Authors:** Yvonne Liu, Johann-Georg Hocher, Shujuan Ma, Liang Hu, Huijun Chen, Xiaoli Zhang, Fei Gong, Bernhard K. Krämer, Ge Lin, Berthold Hocher

**Affiliations:** 1 Fifth Department of Medicine (Nephrology/Endocrinology/Rheumatology/Pneumology), University Medical Centre Mannheim, University of Heidelberg, 68167 Mannheim, Germany; 2 Reproductive and Genetic Hospital of CITIC-Xiangya, Changsha, 410008 China; 3 Medical Faculty of Charité Universitätsmedizin Berlin, 10117 Berlin, Germany; 4 Second Faculty of Medicine, Charles University, Prague, 150 06 Czech Republic; 5 Institute of Reproductive and Stem Cell Engineering, School of Basic Medical Science, Central South University, Changsha, 410017 China; 6 Key Laboratory of Stem Cells and Reproductive Engineering, Ministry of Health, Changsha, 410017 China; 7 Center for Development and Regeneration, Guangzhou Institutes of Biomedicine and Health, Chinese Academy of Sciences, Guangzhou, 510530 China; 8 Centre for Preventive Medicine and Digital Health Baden Württemberg (CPDBW), Medical Faculty Mannheim, University of Heidelberg, 68167 Mannheim, Germany; 9 European Centre for Angioscience ECAS, Medical Faculty Mannheim, University of Heidelberg, 68167 Mannheim, Germany; 10 Institute of Medical Diagnostics, IMD, 12247 Berlin, Germany

**Keywords:** Assisted reproductive technologies, In vitro fertilization, Dyslipidemia, Gestational diabetes mellitus, Ldl hdl ratio

## Abstract

**Background & Objective:**

To analyze whether there is an association between pre-pregnancy lipid parameters and gestational diabetes mellitus (GDM) in women undergoing assisted reproductive technologies (ART), a group especially at risk for GDM, and if so, which parameter is associated the strongest.

**Methods:**

Data was collected at the Reproductive and Genetic Hospital CITIC-Xiangya in Changsha, China from January 2017 to December 2018. The measured lipid parameters include LDL (low-density lipoprotein), HDL (high-density lipoprotein), TC (total cholesterol), and TG (triglycerides).

**Results:**

119 (15.5%) of the 767 patients developed GDM. On average, women who developed GDM were older, had a higher BMI, LDL, TC, and TG, and lower HDL. After adjusting for confounders, LDL and HDL showed a significant association with GDM (*p* < 0.05), but TC and TG did not. Binary LDL/HDL and TC/HDL ratios showed the strongest association with GDM incidence (OR 1.957 [95%CI 1.258–3.044] and 1.942 [1.243–3.034] respectively). Subgroup analysis showed that an elevated LDL/HDL ratio also increased GDM risk in subgroups with a typically lower prevalence of GDM, such as young women with a low BMI and low blood pressure. Both lipid ratios (LDL/HDL and TC/HD) show strong interactions with baseline age, fasting plasma glucose, and LH.

**Conclusions:**

In this cohort of Chinese women undergoing ART, pre-pregnancy LDL/HDL and TC/HDL were associated with GDM the strongest from the lipid parameters and could be useful to estimate GDM risk even before ART treatments and pregnancy.

**Clinical trial number:**

NCT03503006 registered on the 21st of March 2018 (on clinicaltrials.gov). https://clinicaltrials.gov/study/NCT03503006?locStr=Changsha,%20Hunan,%20China&country=China&state=Hunan&city=Changsha&cond=ivf&rank=2.

**Supplementary Information:**

The online version contains supplementary material available at 10.1186/s12958-024-01320-9.

## Introduction

With the change in diet and an increasingly sedentary lifestyle, obesity and high lipid levels are becoming more and more prevalent. Especially China, which in the past had relatively low rates of dyslipidemia, has seen an increase to more than one-third of the general adult population [1]. Dyslipidemia can be diagnosed according to different lipid parameters: LDL (low-density lipoproteins), HDL (high-density lipoproteins), total cholesterol (TC), and/or triglycerides (TG).

Several studies have shown an association between dyslipidemia and gestational diabetes mellitus (GDM) [2, 3], which is one of the most common pregnancy complications with a comparably high incidence in Southeast Asia [4]. Another population group with an increased risk of developing GDM are women undergoing ART (assisted reproductive technologies), for which there are different hypotheses, including the influence of hormonal therapies, as well as the factor that women undergoing ART commonly have certain baseline characteristics or diagnoses causing infertility and associated with GDM such as a higher prevalence of PCOS compared to women conceiving naturally [5, 6]. In the last decades, the use of ART has significantly increased and has given more and more infertile couples the chance to have their own child [7]. From a scientific perspective, it also offers a unique opportunity to follow the course of a pregnancy before it even occurs.

Because GDM is such a common pregnancy complication with many short- and long-term complications for mother and child [8], it is important to assess potential risk factors. By identifying high-risk patients, it would be possible to take early preventative measures. To date, more attention has been paid to the effects of maternal age and body mass index (BMI) on GDM [9]. There have also been studies on the effects of lipid parameters but to a much lesser extent.

Additionally, these findings have not been studied in women undergoing in-vitro fertilization (IVF) or intracytoplasmic sperm injection (ICSI), which is a population especially at risk for GDM. Therefore, this study aims to and is the first to investigate the association between pre-pregnancy lipid levels and GDM in this specific cohort. Our objective is firstly, to identify whether and which lipid parameters are associated with GDM in IVF/ICSI patients and therefore which parameter is the most suitable for tracking lipid status, even before pregnancy, to identify patients with a high-risk profile for this pregnancy complication.

## Methods

### Study design

Data for this retrospective study was collected in a time period of two years from January 2017 to December 2018 at the Reproductive and Genetic Hospital CITIC-Xiangya in Changsha, China. The primary study focused on the effect of vitamin D levels on IVF/ICSI outcomes [10]. The study was conducted according to the *Declaration of Helsinki for Medical Research involving Human Subjects* and was approved by the hospital’s ethics committee (approval number: LL-SC-2018-014). All patients gave their informed consent prior to study participation.

### Study population

From a total of 2569 patients included in the initial study, 767 patients were included in this analysis, as patients who did not achieve a successful pregnancy and those with incomplete data regarding lipids were excluded. All participants underwent their first IVF/ICSI treatment, were all primigravida, and were aged between 18 and 40 years. Patients were excluded, as previously described [10], in any of the following cases: [1] if they had received oocyte donation, 2) uterine malformation, 3) endometriosis, 4) uterine adhesions, 5) untreated hydrosalpinx, 6) uterine myoma, 7) Cushing syndrome, 8) adult-onset adrenogenital syndrome, 9) hypothalamic or pituitary disease causing infertility, 10) diabetes mellitus type 1 or 2 prior to pregnancy, or 11) hypertension prior to pregnancy. Further details on patient selection can be seen in Supplementary Fig. 1.

### Measurement of study end points

The lipid parameters (LDL, HDL, TC, and TG) were measured at baseline in a fasting state – before pregnancy and any IVF/ICSI procedures – in the central hospital laboratory as part of the routine evaluation of the patients. All clinical chemical analyses in this laboratory are part of strict quality controls for clinical chemistry according to the laws of the People’s Republic of China. In addition, we also calculated lipid ratios, which are not routinely assessed in a clinical context but may be better indicators of lipid status than measured lipid parameters [11]. During IVF, patients receive ovarian hyperstimulation, after which oocytes are retrieved, cultured, and inseminated (if necessary, using ICSI). The created embryos are cultured and then transferred into the uterus.

If a successful pregnancy was achieved, the patients were followed up during and after their pregnancy through self-reports and phone calls. All patients underwent a GDM screening (oral glucose tolerance test) during their pregnancy. GDM was diagnosed according to the IADPSG (International Association of Diabetes in Pregnancy Study Groups) 2010 guidelines [12]: if the measured plasma glucose was either 1) ≥ 5.1mmol/L (92 mg/dL) before, 2) ≥ 10.0mmol/L (180 mg/dL) one hour after, or 3) ≥ 8.5mmol/L (153 mg/dL) two hours after taking a 75 g glucose solution.

### Statistical analysis

To test for normality, we created histograms and used the Kolmogorov-Smirnov as well as the Shapiro-Wilk test. Patient characteristics are represented as frequency (%) or median (interquartile range, IQR). To test for a significant difference in population characteristics between GDM and non-GDM patients, we used the Chi-square (χ^2^) test for categorical variables and the Mann-Whitney U test for continuous variables. Multivariate logistic regression considered all significant factors from Table 1 (age, BMI, fasting plasma glucose, LH, and estrogen) in Model A and risk factors for GDM from the literature in Model B (age, BMI, fasting plasma glucose measured before embryo transfer, multiparity, total testosterone, antral follicle count (AFC), anti-Müllarian hormone (AMH), and vitamin D). Since we did not have documentation of the presence of polycystic ovary syndrome (PCOS), which are patients with an elevated risk of GDM [13], we included three surrogate parameters in the multivariate regression: total testosterone, which is a biochemical parameter for hyperandrogenism, one of the main diagnostic criteria of PCOS [14, 15], AFC, which corresponds to the polycystic ovarian morphology (PCOM), another criterion of PCOS, and AMH, which is a biochemical parameter that is often increased in women with PCOS as it correlates with PCOM [16]. We also included vitamin D in the analysis, as some studies have also found a correlation between low vitamin levels and GDM [17, 18]; however, the association is uncertain as there have been contrasting findings [19, 20].

Receiver operating characteristic (ROC) analysis was used to determine optimal cut-off values for binary analysis. Furthermore, we created a forest plot to represent the results of subgroup analyses and 3D plots to show interactions between lipids and other risk factors for GDM.

**Fig. 1:**
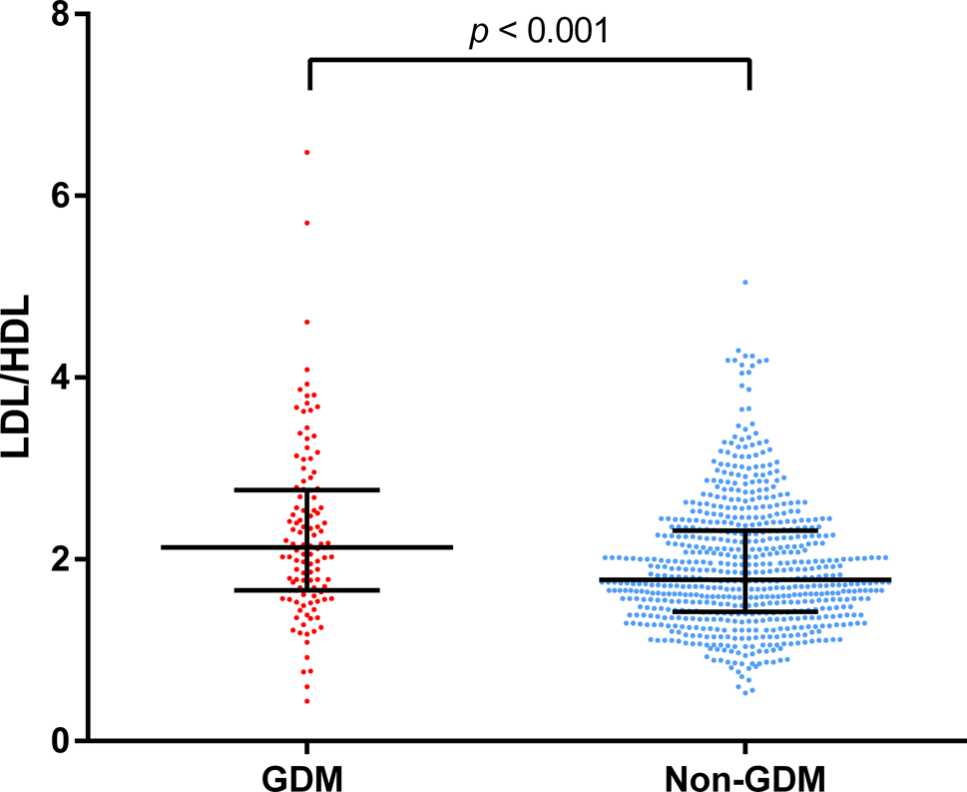
LDL/HDL levels in GDM versus non-GDM patients. Plots are presented as median and IQR (interquartile range). *p*-values were calculated with the Mann-Whitney U test. *Abbrevations* GDM = gestational diabetes mellitus; LDL = low-density lipoproteins; HDL = high-density lipoproteins. Plots for other lipid parameters can be seen in Supplementary Fig. 4a-f

**Fig. 2:**
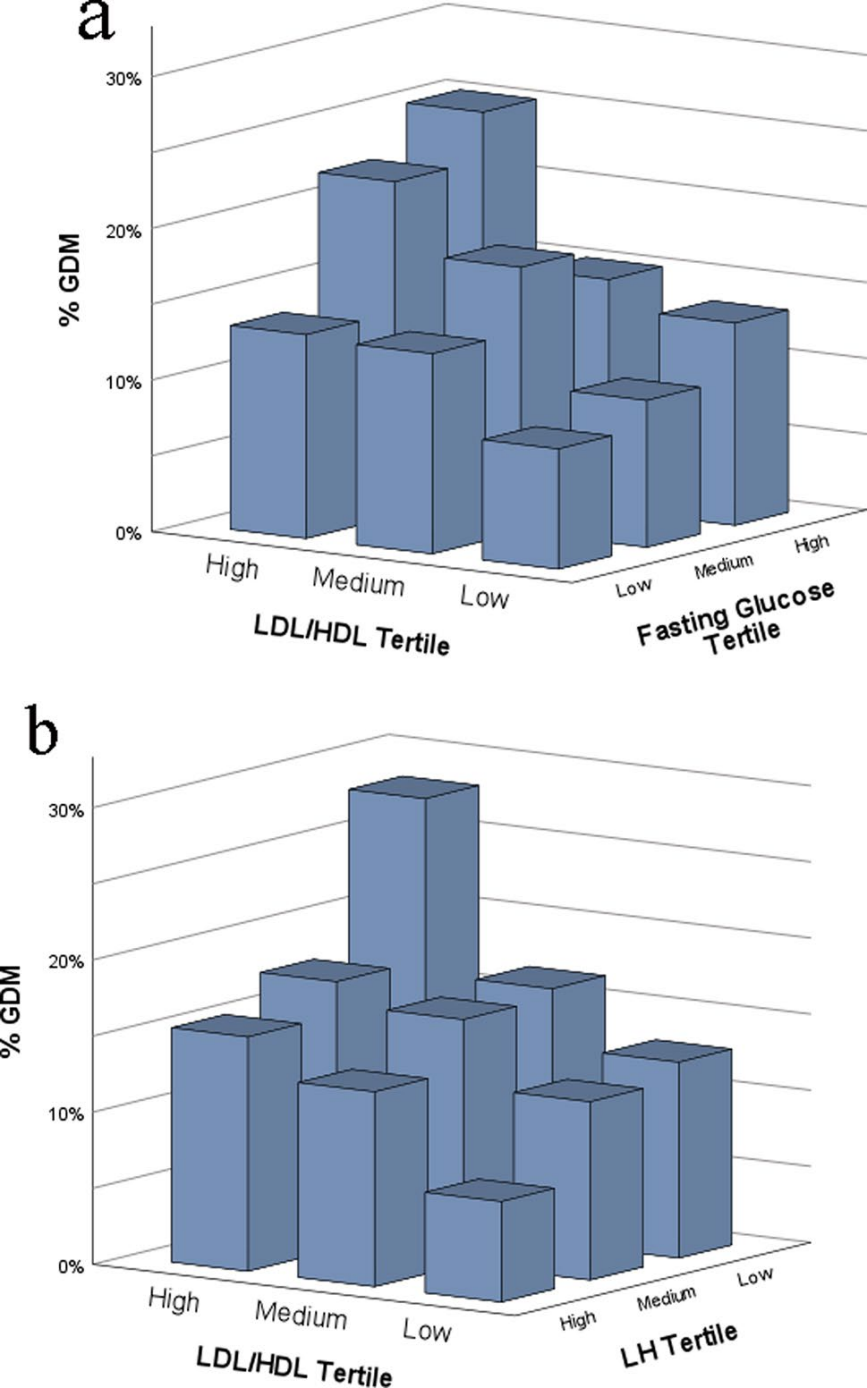
3D Plots – Effects of LDL/HDL ratio and other GDM risk factors on GDM incidence in women undergoing IVF/ICSI. LDL/HDL ratio is displayed with fasting plasma glucose (**a**) and LH (**b**). Comparing the group’s lowest vs. highest tertile of LDL/HDL ratio and blood glucose, 7.9% developed GDM in the lowest and 25.5% in the highest group (*p* < 0.001). Women in the lowest LDL/HDL tertile and highest LH tertile had a GDM rate of 6.6%, whilst women in the highest LDL/HDL tertile and lowest LH tertile had a GDM rate of 28.2% (*p* < 0.001). *Abbreviations* GDM = gestational diabetes mellitus; E2 = estradiol; ICSI = intracytoplasmic sperm injection; IVF = in-vitro fertilization; LDL = low-density lipoproteins; LH = luteinizing hormone; HDL = high-density lipoproteins. 3D Plots for age, BMI, and estradiol can be seen in Supplementary Fig. 2a-c

**Fig. 3:**
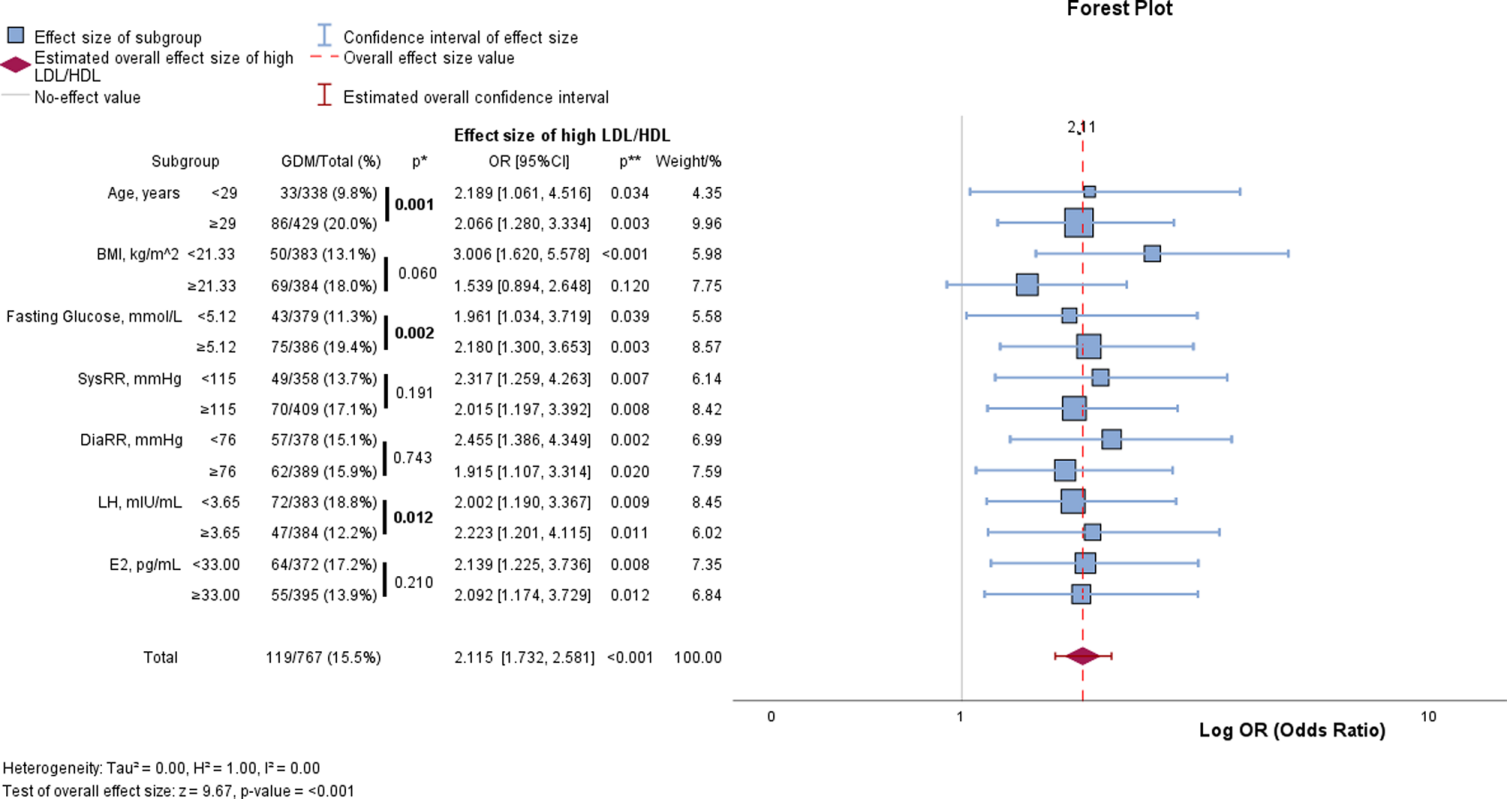
Forest plot – subgroup analysis of GDM prevalence and the association between LDL/HDL ratio and GDM. The first part of the table shows the prevalence of GDM in each of the subgroups. p* = represents the significance of a difference in GDM rate between the subgroups, calculated using the Chi-squared test. OR and p** show the effect size of a high LDL/HDL ratio (cut-off calculated using the Youden-Index) on the GDM rate in each of the subgroups. *Abbreviations* BMI = body mass index; CI = confidence interval; DiaRR = diastolic blood pressure; E2 = estradiol; GDM = gestational diabetes mellitus; LDL = low-density lipoproteins; LH = luteinizing hormone; HDL = high-density lipoproteins; N = no GDM; OR = odds ratio; SysRR = systolic blood pressure

All data was analyzed using SPSS (Statistical Package for Social Sciences), version 29.0 (IBM Corporation, Armonk, New York, USA). We created Fig. 1 using GraphPad Prism 6 (GraphPad Software Inc., San Diego, California, USA) and Figs. 2a-b and 3 using SPSS. The statistical significance level was set to *p* < 0.05.

## Results

Of the 767 patients included in this study, 119 (15.5%) developed GDM during their pregnancy. All measured pre-pregnancy lipid parameters (LDL, HDL, TC, and TG) were not normally distributed (Supplementary Fig. 2a-d), which was also confirmed by the statistical Kolmogorov-Smirnov and Shapiro-Wilk tests for normality (all *p* < 0.001). 15.0% of the patients had elevated LDL levels, 9.0% had low HDL, 8.2% had elevated TC, and 15.5% had elevated TG levels according to the AACE (American Association of Clinical Endocrinologists and American College of Endocrinology) 2017 guidelines [21] and Chinese guidelines [22] (Supplementary Fig. 3). In total, 233 women (30.4%) had at least one pathological lipid measurement to be diagnosed with dyslipidemia.

Detailed characteristics of the study cohort are displayed in Table 1. Overall, the median age was 29.0 years (IQR 27.0–31.0), and the median BMI was 21.33 kg/m^2^ (IQR 19.53–23.01). For the lipid parameters, median LDL was 2.23 mmol/L (IQR 2.66–3.10), median HDL was 1.43 mmol/L (IQR 1.22–1.67), median TC was 4.13 mmol/L (IQR 3.67–4.60), and median TG was 1.00 mmol/L (IQR 0.73–1.14). Table 1 also compares these characteristics between patients with and without the diagnosis of GDM. Women who developed GDM were statistically older (30.0 versus 29.0 years, *p* < 0.001), had a higher BMI (21.91 versus 21.23 kg/m^2^, *p* = 0.005), fasting glucose (5.21 versus 5.10 mmol/L, *p* = 0.002), LDL (2.90 versus 2.62 mmol/L, *p* = 0.004), TG (1.12 versus 0.98 mmol/L, *p* = 0.010), and lower HDL (1.32 versus 1.44 mmol/L, *p* < 0.001), LH (luteinizing hormone) (3.26 versus 3.73 mIU/mL, *p* = 0.003), and estradiol (32.00 versus 33.00 pg/mL, *p* = 0.046).

**Table 1:** Clinical characteristics of study participants by GDM incidence

Parameters	All (*n* = 767)	GDM (*n* = 119)	Non-GDM (*n* = 648)	*p*-Value
Age, years	29.0 (27.0–31.0)	30.0 (28.0–33.0)	29.0 (27.0–31.0)	**< 0.001**
Weight, kg	53.00 (48.00–58.00)	54.50 (49.00–60.00)	53.00 (48.00–58.00)	0.142
BMI, kg/m^2^	21.33 (19.53–23.01)	21.91 (20.20-23.74)	21.23 (19.44–22.86)	**0.005**
TC, mmol/L	4.13 (3.67–4.60)	4.25 (3.74–4.76)	4.10 (3.65–4.57)	0.059
LDL, mmol/L	2.23 (2.66–3.10)	2.90 (2.31–3.30)	2.62 (2.21–3.09)	**0.004**
HDL, mmol/L	1.43 (1.22–1.67)	1.32 (1.12.1.55)	1.44 (1.23–1.68)	**< 0.001**
TG, mmol/L	1.00 (0.73–1.41)	1.12 (0.78–1.60)	0.98 (0.72–1.38)	**0.010**
Fasting glucose, mmol/L	5.12 (4.87–5.37)	5.21 (4.99–5.49)	5.10 (4.86–5.35)	**0.002**
Gestational hypertension, *n* (%)	26 (3.4%)	5 (4.2%)	21 (3.2%)	0.594
Systolic RR, mmHg	115 (108–121)	117 (109–124)	115 (108–121)	0.200
Diastolic RR, mmHg	76 (70–81)	77 (70–81)	76 (70–81)	0.308
AMH, ng/mL	6.23 (4.06–10.28)	5.24 (3.73–10.62)	6.49 (4.19–10.28)	0.096
FSH, mIU/mL	5.64 (4.81–6.64)	5.51 (4.58–6.42)	5.69 (4.85–6.66)	0.191
LH, mIU/mL	3.65 (2.59–5.09)	3.26 (2.29–4.46)	3.73 (2.67–5.13)	**0.003**
Estradiol, pg/mL	33.00 (27.00–43.00)	32.00 (24.00–40.00)	33.00 (27.00–43.00)	**0.046**
Testosterone, ng/mL	0.27 (0.21–0.34)	0.27 (0.20–0.34)	0.27 (0.21–0.34)	0.789
AFC	15.00 (12.00–21.00)	14.00 (11.00–20.00)	15.00 (12.00–22.00)	0.109
Infertility type				0.787
1°	449 (58.5%)	71 (59.7%)	378 (58.3%)	
2°	318 (41.5%)	48 (40.3%)	270 (41.7%)	
Fertilization method				0.459
IVF	509 (66.4%)	84 (70.6%)	425 (65.6%)	
ICSI	132 (17.2%)	16 (13.4%)	116 (17.9%)	
IVF + ICSI	126 (16.4%)	19 (16.0%)	107 (16.5%)	
Pregnancy type				0.332
Singleton	496 (64.7%)	412 (63.6%)	84 (70.6%)	
Multiple	262 (34.2%)	227 (35.0%)	35 (29.4%)	
Delivery method				0.337
Normal	213 (27.8%)	36 (30.3%)	177 (27.3%)	
Cesarean	544 (70.9%)	83 (69.7%)	461 (71.1%)	

Data is presented as frequency, *n* (%) or median (IQR, interquartile range). *p*-values were calculated with the Chi-square (χ^2^) test for categorical variables and the Mann-Whitney U test for continuous variables. Abbreviations: AFC = antral follicle count; AMH = anti-Müllarian hormone; BMI = body mass index; GDM = gestational diabetes mellitus; FSH = follicle-stimulating hormone; HDL = high-density lipoproteins; ICSI = intracytoplasmic sperm injection; IVF = in-vitro fertilization; LDL = low-density lipoproteins; LH = luteinizing hormone; RR = blood pressure; TC = total cholesterol; TG = triglycerides

Figure 1 graphically illustrates the comparison of LDL/HDL between GDM and non-GDM patients and Supplementary Fig. 4a-f further show this for measured lipid parameters and other lipid ratios, including TC/HDL and TG/HDL. Along with LDL, HDL, and TG, all three lipid ratios show a significant difference between the two groups (*p* < 0.05), whilst TC was not statistically different (*p* = 0.059).

In further analysis, we performed multivariate logistic regression, creating Model A (considering confounders that were statistically significant in Table 1: age, BMI, fasting plasma glucose, LH, and estradiol) and Model B (considering confounders previously described in literature [8]: age, BMI, fasting plasma glucose, multiparity, total testosterone, AFC, AMH, and total vitamin D). Both models showed that continuous LDL, HDL, LDL/HDL ratio, and TC/HDL ratio were still significantly different in GDM versus non-GDM patients (Table 2). The ratios LDL/HDL and TC/HDL displayed higher odds ratios (OR) (OR 1.628 for LDL/HDL and 1.452 for TC/HDL in Model A), as well as more significant *p*-values (*p* < 0.001) than continuous LDL (OR 1.351 and *p* = 0.035 in Model A). HDL, which is inversely proportional to GDM incidence, also still showed a significant difference between GDM and non-GDM patients (OR 0.431, *p* = 0.013 in Model A). However, the other lipid parameters, TC, TG, and TG/HDL ratio, were not significantly associated with GDM after adjustment for confounders (*p* = 0.171, 0.341, and 0.253 in Model A, respectively). Full tables of the multivariate regression can be seen in Supplementary Tables 1–5.

**Table 2:** Multivariate logistic regression for lipid parameters and GDM incidence

	B	OR	95% CI	Standard Error	*p*-Value
**LDL**					
***Continuous LDL***					
Model A	0.301	1.351	[1.021, 1.788]	0.143	**0.035**
Model B	0.404	1.499	[1.074, 2.091]	0.170	**0.017**
***Binary LDL****(cut-off 2.95mmol/L)*					
Model A	0.594	1.812	[1.196, 2.745]	0.212	**0.005**
Model B	0.710	2.033	[1.255, 3.293]	0.246	**0.004**
**HDL**					
***Continuous HDL***					
Model A	-0.842	0.431	[0.221, 0.839]	0.340	**0.013**
Model B	-0.991	0.371	[0.168, 0.821]	0.405	**0.014**
***Binary HDL****(cut-off 1.32mmol/L)*					
Model A	-0.593	0.553	[0.355, 0.860]	0.226	**0.009**
Model B	-0.619	0.539	[0.321, 0.902]	0.263	**0.019**
**LDL/HDL**					
***Continuous LDL/HDL***					
Model A	0.487	1.628	[1.244, 2.130]	0.137	**< 0.001**
Model B	0.560	1.750	[1.286, 2.383]	0.157	**< 0.001**
***Binary LDL/HDL****(cut-off 2.02)*					
Model A	0.671	1.957	[1.258, 3.044]	0.225	**0.003**
Model B	0.782	2.186	[1.308, 3.656]	0.262	**0.003**
**TC/HDL**					
***Continuous TC/HDL***					
Model A	0.373	1.452	[1.164, 1.811]	0.113	**< 0.001**
Model B	0.429	1.536	[1.196, 1.973]	0.128	**< 0.001**
***Binary TC/HDL****(cut-off 3.15)*					
Model A	0.664	1.942	[1.243, 3.034]	0.228	**0.004**
Model B	0.817	2.264	[1.346, 3.807]	0.265	**0.002**
**TG/HDL**					
***Continuous TG/HDL***					
Model A	0.119	1.126	[0.919, 1.381]	0.104	**0.253**
Model B	0.148	1.160	[0.927, 1.451]	0.114	**0.194**
***Binary TG/HDL****(cut-off 0.68)*					
Model A	0.537	1.710	[1.104, 2.649]	0.223	**0.016**
Model B	0.731	2.076	[1.240, 3.475]	0.263	**0.005**

*p*-values were calculated using multivariate logistic regression. Binary cut-offs were determined with ROC analysis. Model A: adjusted to all significant factors in Table 1 (age, BMI, pre-pregnancy fasting plasma glucose, LH, and E2); Model B: adjusted to risk factors from literature (age, BMI, pre-pregnancy fasting plasma glucose, multiparity, total testosterone, antral follicle count, anti-Müllarian hormone, and total vitamin D). Abbreviations: B = Regression coefficient; BMI = body mass index; CI = Confidence Interval for OR; GDM = gestational diabetes mellitus; HDL = high-density lipoproteins; LDL = low-density lipoproteins; LH = luteinizing hormone; OR = odds ratio; ROC = receiver operating characteristic; TC = total cholesterol; TG = triglycerides. The full tables can be found in Supplementary Tables 1–5

To find an optimal cut-off value, we performed ROC analyses and calculated the Youden-Index. The cut-off values were as follows: LDL 2.95 mmol/L, HDL 1.32 mmol/L, LDL/HDL 2.02, and TC/HDL 3.15. In binary multivariate logistic regression, these cut-offs were shown to have better odds ratios – for LDL (OR 1.812 in Model A) and the lipid ratios (OR LDL/HDL 1.957; TC/HDL 1.942; TG/HDL 1.710), but not for HDL (OR 0.553) (Table 2).

The 3D plots (Fig. 2a-b and Supplementary Fig. 5a-c) further show that the pre-pregnancy LDL/HDL ratio is strongly related to GDM, but also show interactions with other risk factors for GDM identified in Table 1. The data shows that 7.9% of the women developed GDM if they were in the lowest tertiles for LDL/HDL ratio and fasting glucose, whilst 25.5% developed GDM if they were in the highest tertiles for both categories. 6.6% of women in the lowest tertile for LDL/HDL ratio and the highest tertile of LH received the diagnosis of GDM, compared to 28.2% of women in the highest LDL/HDL ratio tertile and lowest LH tertile. Statistically, this was also confirmed using the Mann-Whitney U test, showing a significant difference in GDM-rates (*p* < 0.001) in both cases. This shows that the interaction between these factors significantly affects GDM risk.

Analysis of subgroups (displayed in Fig. 3) showed that there was a higher prevalence of GDM in women above the age of 29 years, with a high BMI, high fasting plasma glucose, and blood pressure. The rate of GDM was 9.8% in women aged < 29 years compared to 20.0% in women aged ≥ 29 years. However, the odds ratios show that an elevated LDL/HDL ratio also had a relevant effect on GDM occurrence in women who were younger, had a lower BMI, and had lower blood pressure.

## Discussion

From the measured lipid parameters, LDL and HDL were significantly different in GDM vs. non-GDM patients even after adjustment for confounders. The LDL/HDL and TC/HDL ratios were most significantly associated with the incidence of GDM in women undergoing IVF/ICSI.

119 (15.5%) of the 767 patients were diagnosed with GDM. This corresponds to the results of a large-scale meta-analysis, which estimated China’s overall GDM rate to be at 14.8% [23]. However, there are significant regional differences: several similar studies with pregnant women in different cities of China found the prevalence of GDM to be at 15.2% [24] and 19.7% in Beijing [25], and 10.5% in Shanghai [26]. Concerning lipid parameters, the prevalence of dyslipidemia amongst adults in China has been estimated to be 34.0%, though it is significantly higher in men and older people [1]. In this cross-sectional analysis, the average age of the participants was 50.24 years, and the rates of dyslipidemia were significantly higher in men (41.9%) than in women (32.5%). In our cohort of women undergoing IVF/ICSI, 30.4% of the study participants had at least one lipid parameter outside the recommended values of the AACE 2017 [21] and Chinese guidelines from 2016 [22], which is comparable to observed dyslipidemia rates in China.

Recorded factors that were significantly associated with GDM were age, BMI, fasting glucose, LH, and E2 (all *p* < 0.05). Age and BMI are known from the literature as risk factors for GDM [8], as well as hyperglycemia before pregnancy, which is represented by the pre-pregnancy fasting glucose measurement. One study suggested that the FSH/LH ratio could be an early predictor of GDM for women undergoing IVF [27]. A possible explanation could be the relationship between LH and estrogen, whereby the latter is known to be a protective factor against diabetes and modify insulin resistance [28]. Low levels of SHBG (sex hormone binding globulin), which are also closely interrelated with LH levels, are also associated with increased insulin resistance and therefore with the occurrence of GDM [29]. However, the exact mechanisms remain to be further explored.

From the initial univariate analysis, three of the four measured lipid parameters, LDL, HDL, and TG, were significantly different in GDM versus non-GDM patients. Of these, HDL showed the strongest association (*p* < 0.001), whilst TC was not significantly different (*p* = 0.059). After adjusting for confounding factors, TG also did not show a significant association with GDM (in both Models A and B).

The lipid ratios LDL/HDL and TC/HDL showed strong associations with GDM incidence, even after adjustment for confounders. Looking at binary LDL/HDL for example, it showed a significant odds ratio (OR 1.957 [95%CI 1.258, 3.044], even higher than for BMI with GDM (Supplementary Tables 3a-d)). Our findings suggest that analyzing lipid ratios, along with other known risk factors such as age, baseline fasting glucose, and LH, could be a cost-efficient method to identify women undergoing IVF/ICSI who have a high risk for developing GDM.

Previous studies that also measured the same four lipid parameters as in our study, but in a normally conceived pregnancy, have found that TG and HDL were significantly associated with GDM, but not TC and LDL [2, 30]. Another study in China by Shen et al. [26] found that whilst TG was associated with GDM throughout pregnancy, TC and LDL were only higher in the first trimester in women who developed GDM. This indicates how lipid parameters also significantly change throughout the course of pregnancy. Even though our analysis showed differing results, whereby TG was only significantly associated with GDM in univariate analysis but not after adjustment for confounders, we also found that there was a strong, inverse association with HDL, as well as a positive association with LDL. In addition, many studies did not assess lipid ratios, which, in our study, had the strongest statistical association with GDM. For further studies, it would be meaningful to include calculated lipid ratios, as they may be a better indicator of lipid status [11].

The subgroup analysis in our cohort confirmed that older women, women with a higher BMI, higher pre-pregnancy fasting plasma glucose, blood pressure, as well as low LH showed a higher prevalence of GDM. As seen in Fig. 3, the LDL/HDL ratio also had a significant effect on GDM risk in women who were younger, with a lower BMI, and lower blood pressure. Contrastingly, O’Malley et al. found that the association between dyslipidemia and GDM was only present in patients with obesity [3]. Our study indicates that there might be a high significance of lipid levels in our study cohort of Chinese women undergoing IVF/ICSI and in women with a low risk of GDM. While the median BMI in our study cohort was well within the normal range at 21.33 kg/m^2^, O’Malley’s study focused on women with at least one risk factor for GDM and the cohort had an overall obesity rate of 57.4%.

The 3D plots (Fig. 2a-b and Supplementary Fig. 5a-c), which display the relationship between LDL/HDL with other GDM risk factors on the incidence of GDM, show that in our cohort, the LDL/HDL ratio was an even stronger predictor for GDM incidence than BMI. However, the analyzed factors also interact with each other, meaning that there are complex underlying mechanisms related to the development of GDM. For example, women in our cohort with a high LDL/HDL ratio and a high pre-pregnancy fasting glucose level had a much higher risk of developing GDM, compared to women with low LDL/HDL and fasting glucose (Fig. 2a).

Concerning the association between lipids and GDM development, there are several explanations and hypotheses, but the exact mechanisms remain unclear. Eppel et al. found that high levels of TG were associated with insulin resistance as well as β-cell dysfunction [31], which are both factors that play a significant role in the pathogenesis of GDM. In combination, impaired β-cells cannot compensate for the increased insulin resistance, thus leading to higher plasma glucose levels and the development of diabetes. Another mechanism is an alteration in the lipid metabolism during pregnancy, whereby lipid levels increase at the beginning of pregnancy as a result of the anabolic metabolism and then are broken down again [32]; increased fatty acids and glycerol are transferred from the breakdown of adipose tissue to the placenta as well as to the liver for gluconeogenesis [33] – thus, producing more glucose and leading to GDM. Another case-control study found that women who developed GDM had lower pre-pregnancy LDL peak diameter size as well as lower HDL levels [34]. Smaller LDL particles are more prone to oxidation, leading to β-cell dysfunction, which could also contribute to increased GDM risk. Further research into the exact pathomechanisms is required to fully understand how lipids contribute to the development of GDM, especially in women undergoing IVF/ICSI, a subgroup of women with a very high GDM risk.

There are several study limitations, including that the family history of diabetes, the diagnosis of PCOS, and the use of medication, which are important risk factors for GDM, as well as pre-pregnancy HbA1c, were not recorded because this was a retrospective analysis of a previously prospective study focusing on IVF/ICSI outcomes. However, we have corrected for the biochemical diagnostic criteria for PCOS in the form of total testosterone concentration, as well as AFC and AMH in Model B of the multivariate logistic regression. Another limitation is that baseline blood tests were performed at different times before conception. As lipid levels can be significantly influenced by lifestyle factors such as diet and physical activity as well as uncontrollable factors, it could be beneficial to have several measurements from each study individual and evaluate their average. Furthermore, it is important to note that our study was a single-center study with only Chinese women, which is important because GDM has different prevalences in different regions and ethnicities. Therefore, the findings need to be studied in other cohorts to generalize our conclusions.

Strengths of this study include that this is the first study focusing on the effects of pre-pregnancy lipid levels on GDM incidence specifically in women undergoing ART. Furthermore, we measured four different lipid parameters and compared them, in addition to three different lipid ratios.

## Conclusions

From all lipid measurements and ratios, pre-pregnancy LDL/HDL and TC/HDL ratios were most significantly associated with GDM incidence in this post-hoc analysis of women undergoing IVF/ICSI. Analysis of pre-pregnancy LDL/HDL and TC/HDL might be a novel and cost-effective tool to identify women at high GDM risk in daily clinical practice in IVF centers.

## Electronic supplementary material

Below is the link to the electronic supplementary material.

Supplementary Material 1

Supplementary Material 2

## Data Availability

The original contributions presented in the study are included in the article/supplementary material, further inquiries can be directed to the corresponding author.

## Abbreviations

AFC: Antral follicle count
AMH: Anti-Müllarian hormone
ART: Assisted reproductive technology
BMI: Body mass index
E2: Estradiol
GDM: Gestational diabetes mellitus
HDL: High-density lipoproteins
ICSI: Intracytoplasmic sperm injection
IVF: In-vitro fertilization
LDL: Low-density lipoproteins
LH: Luteinizing hormone
TC: Total cholesterol
TG: Triglycerides

## References

[1] Pan L, Yang Z, Wu Y, Yin RX, Liao Y, Wang J, et al. The prevalence, awareness, treatment and control of dyslipidemia among adults in China. Atherosclerosis. 2016;248:2–9. 26978581 10.1016/j.atherosclerosis.2016.02.006

[2] Ryckman KK, Spracklen CN, Smith CJ, Robinson JG, Saftlas AF. Maternal lipid levels during pregnancy and gestational diabetes: a systematic review and meta-analysis. BJOG. 2015;122(5):643–51. 25612005 10.1111/1471-0528.13261

[3] O’Malley EG, Reynolds CME, Killalea A, O’Kelly R, Sheehan SR, Turner MJ. Maternal obesity and dyslipidemia associated with gestational diabetes mellitus (GDM). Eur J Obstet Gynecol Reprod Biol. 2020;246:67–71. 31962258 10.1016/j.ejogrb.2020.01.007

[4] Wang H, Li N, Chivese T, Werfalli M, Sun H, Yuen L, et al. IDF Diabetes Atlas: estimation of Global and Regional Gestational Diabetes Mellitus Prevalence for 2021 by International Association of Diabetes in Pregnancy Study Group’s Criteria. Diabetes Res Clin Pract. 2022;183:109050. 34883186 10.1016/j.diabres.2021.109050

[5] Ashrafi M, Gosili R, Hosseini R, Arabipoor A, Ahmadi J, Chehrazi M. Risk of gestational diabetes mellitus in patients undergoing assisted reproductive techniques. Eur J Obstet Gynecol Reprod Biol. 2014;176:149–52. 24630294 10.1016/j.ejogrb.2014.02.009

[6] Bosdou JK, Anagnostis P, Goulis DG, Lainas GT, Tarlatzis BC, Grimbizis GF, et al. Risk of gestational diabetes mellitus in women achieving singleton pregnancy spontaneously or after ART: a systematic review and meta-analysis. Hum Reprod Update. 2020;26(4):514–44. 32441298 10.1093/humupd/dmaa011 PMC7317285

[7] Kushnir VA, Smith GD, Adashi EY. The future of IVF: the New Normal in Human Reproduction. Reprod Sci. 2022;29(3):849–856. 10.1007/s43032-021-00829-3 PMC8722744 34981459

[8] Chiefari E, Arcidiacono B, Foti D, Brunetti A. Gestational diabetes mellitus: an updated overview. J Endocrinol Invest. 2017;40(9):899–909. 28283913 10.1007/s40618-016-0607-5

[9] Yong HY, Shariff ZM, Yusof BNM, Rejali Z, Tee YY, Bindels J et al. Independent and combined effects of age, body mass index and gestational weight gain on the risk of gestational diabetes mellitus. Sci Rep. 2020;10. 10.1038/s41598-020-65251-2 PMC7244566 32444832

[10] Cai S, Li J, Zeng S, Hu L, Peng Y, Tang S, et al. Impact of vitamin D on human embryo implantation-a prospective cohort study in women undergoing fresh embryo transfer. Fertil Steril. 2021;115(3):655–64. 33039126 10.1016/j.fertnstert.2020.09.005

[11] Sheng G, Kuang M, Yang R, Zhong Y, Zhang S, Zou Y. Evaluation of the value of conventional and unconventional lipid parameters for predicting the risk of diabetes in a non-diabetic population. J Transl Med. 2022;20(1):266. 35690771 10.1186/s12967-022-03470-z PMC9188037

[12] International Association of D, Pregnancy Study Groups, Consensus P, Metzger BE, Gabbe SG, Persson B, Buchanan TA, et al. International association of diabetes and pregnancy study groups recommendations on the diagnosis and classification of hyperglycemia in pregnancy. Diabetes Care. 2010;33(3):676–82. 20190296 10.2337/dc09-1848 PMC2827530

[13] Wang Y, Zhao X, Zhao H, Ding H, Tan J, Chen J, et al. Risks for gestational diabetes mellitus and pregnancy-induced hypertension are increased in polycystic ovary syndrome. Biomed Res Int. 2013;2013:182582. 24371816 10.1155/2013/182582 PMC3859212

[14] Joham AE, Norman RJ, Stener-Victorin E, Legro RS, Franks S, Moran LJ, et al. Polycystic ovary syndrome. Lancet Diabetes Endocrinol. 2022;10(9):668–80. 35934017 10.1016/S2213-8587(22)00163-2

[15] Lizneva D, Suturina L, Walker W, Brakta S, Gavrilova-Jordan L, Azziz R. Criteria, prevalence, and phenotypes of polycystic ovary syndrome. Fertil Steril. 2016;106(1):6–15. 27233760 10.1016/j.fertnstert.2016.05.003

[16] Bani Mohammad M, Majdi Seghinsara A. Polycystic ovary syndrome (PCOS), Diagnostic Criteria, and AMH. Asian Pac J Cancer Prev. 2017;18(1):17–21. 28240001 10.22034/APJCP.2017.18.1.17 PMC5563096

[17] Zhang C, Qiu C, Hu FB, David RM, van Dam RM, Bralley A, et al. Maternal plasma 25-hydroxyvitamin D concentrations and the risk for gestational diabetes mellitus. PLoS ONE. 2008;3(11):e3753. 19015731 10.1371/journal.pone.0003753 PMC2582131

[18] Soheilykhah S, Mojibian M, Rashidi M, Rahimi-Saghand S, Jafari F. Maternal vitamin D status in gestational diabetes mellitus. Nutr Clin Pract. 2010;25(5):524–7. 20962313 10.1177/0884533610379851

[19] Rizzo G, Garzon S, Fichera M, Panella MM, Catena U, Schiattarella A et al. Vitamin D and gestational diabetes Mellitus: is there a link? Antioxidants. (Basel). 2019;8(11). 10.3390/antiox8110511 PMC6912234 31731439

[20] Liu Y, Hocher JG, Chen H, Hu L, Zhang X, Cai S, et al. The degree of pre-pregnancy vitamin D deficiency is not associated with gestational diabetes in women undergoing ART. J Endocr Soc. 2023;7(12). 10.1210/jendso/bvad140 PMC10681737 38024652

[21] Jellinger PS, Handelsman Y, Rosenblit PD, Bloomgarden ZT, Fonseca VA, Garber AJ, et al. American Association of Clinical Endocrinologists and American College of Endocrinology Guidelines for Management of Dyslipidemia and Prevention of Cardiovascular Disease. Endocr Pract. 2017;23(Suppl 2):1–87. 28437620 10.4158/EP171764.APPGL

[22] Joint committee for guideline r. 2016 Chinese guidelines for the management of dyslipidemia in adults. J Geriatr Cardiol. 2018;15(1):1–29. 29434622 10.11909/j.issn.1671-5411.2018.01.011 PMC5803534

[23] Gao C, Sun X, Lu L, Liu F, Yuan J. Prevalence of gestational diabetes mellitus in mainland China: a systematic review and meta-analysis. J Diabetes Investig. 2019;10(1):154–62. 29683557 10.1111/jdi.12854 PMC6319492

[24] Li G, Kong L, Zhang L, Fan L, Su Y, Rose JC, et al. Early pregnancy maternal lipid profiles and the risk of gestational diabetes Mellitus Stratified for Body Mass Index. Reprod Sci. 2015;22(6):712–7. 25394643 10.1177/1933719114557896 PMC4502803

[25] Zhu H, He D, Liang N, Lai A, Zeng J, Yu H. High serum triglyceride levels in the early first trimester of pregnancy are associated with gestational diabetes mellitus: a prospective cohort study. J Diabetes Investig. 2020;11(6):1635–42. 32281298 10.1111/jdi.13273 PMC7610113

[26] Shen H, Liu X, Chen Y, He B, Cheng W. Associations of lipid levels during gestation with hypertensive disorders of pregnancy and gestational diabetes mellitus: a prospective longitudinal cohort study. BMJ Open. 2016;6(12):e013509. 28011814 10.1136/bmjopen-2016-013509 PMC5223699

[27] Coussa A, Hasan HA, Barber TM. Early predictors of Gestational Diabetes Mellitus in IVF-Conceived pregnancies. Endocr Pract. 2021;27(6):579–85. 34120700 10.1016/j.eprac.2020.10.020

[28] Paoli MP, Zakharia A, Werstuck GH. The role of estrogen in insulin resistance: a review of clinical and preclinical data. Am J Pathol 2021;9. 10.1016/j.ajpath.2021.05.011 34102108

[29] Hedderson MM, Xu F, Darbinian J, Quesenberry CP, Sridhar S, Kim C et al. Prepregnancy SHBG concentrations and risk for subsequently developing gestational diabetes Mellitus. Diabetes Care. 2014;5. 10.2337/dc13-1965 PMC3994937 24561392

[30] Li Y, Wang X, Jiang F, Chen W, Li J, Chen X. Serum lipid levels in relation to clinical outcomes in pregnant women with gestational diabetes mellitus: an observational cohort study. Lipids Health Dis. 2021;20(1):125. 34587947 10.1186/s12944-021-01565-y PMC8482603

[31] Eppel D, Feichtinger M, Lindner T, Kotzaeridi G, Rosicky I, Yerlikaya-Schatten G, et al. Association between maternal triglycerides and disturbed glucose metabolism in pregnancy. Acta Diabetol. 2021;58(4):459–65. 33387029 10.1007/s00592-020-01644-z PMC8053660

[32] White SL, Pasupathy D, Sattar N, Nelson SM, Lawlor DA, Briley AL, et al. Metabolic profiling of gestational diabetes in obese women during pregnancy. Diabetologia. 2017;60(10):1903–12. 28766127 10.1007/s00125-017-4380-6 PMC6448883

[33] Barrett HL, Dekker Nitert M, McIntyre HD, Callaway LK. Normalizing metabolism in diabetic pregnancy: is it time to target lipids? Diabetes Care. 2014;37(5):1484–93. 24757231 10.2337/dc13-1934

[34] Han ES, Krauss RM, Xu F, Sridhar SB, Ferrara A, Quesenberry CP, et al. Prepregnancy adverse lipid profile and subsequent risk of gestational diabetes. J Clin Endocrinol Metab. 2016;101(7):2721–2727. 10.1210/jc.2015-3904 PMC4929836 27045641

