# Supplementary material for: Pre-pregnancy LDL/HDL and total Cholesterol/HDL ratios are strong predictors of gestational diabetes mellitus in women undergoing assisted reproductive technologies
Source: Reprod Biol Endocrinol. 2024 Dec 5;22:155. doi: 10.1186/s12958-024-01320-9 (PMC11619337; doi:10.1186/s12958-024-01320-9)
Supplement: Supplementary file 1 — Supplementary Material 1 [file 12958_2024_1320_MOESM1_ESM.docx]

**Supplementary Tables**

**Supplementary Tables 1a-d: Multivariate logistic regression for LDL**

Comparisons were made using multivariate logistic regression. AFC = antral follicle count; AMH = anti-Müllarian hormone; B = Regression coefficient; BMI = body mass index; CI = Confidence Interval for OR; E2 = estradiol; GDM = gestational diabetes mellitus; LDL = low-density lipoproteins; LH = luteinizing hormone; OR = odds ratio.

**Supplementary Table 1a:** Multivariate logistic regression for continuous LDL and GDM incidence (Model A)

|  | B | OR | 95% CI | Standard Error | *p*-Value |
| --- | --- | --- | --- | --- | --- |
| LDL continuous | 0.301 | 1.351 | [1.021, 1.788] | 0.143 | **0.035** |
| Age | 0.116 | 1.123 | [1.058, 1.192] | 0.030 | <0.001 |
| BMI | 0.037 | 1.038 | [0.951, 1.134] | 0.045 | 0.403 |
| Plasma glucose | 0.351 | 1.421 | [0.947, 1.134] | 0.207 | 0.090 |
| LH | -0.067 | 0.935 | [0.854, 1.025] | 0.047 | 0.152 |
| E2 | -0.008 | 0.992 | [0.980, 1.004] | 0.006 | 0.205 |

**Supplementary Table 1b:** Multivariate logistic regression for continuous LDL and GDM incidence (Model B)

|  | B | OR | 95% CI | Standard Error | *p*-Value |
| --- | --- | --- | --- | --- | --- |
| LDL continuous | 0.404 | 1.499 | [1.074, 2.091] | 0.170 | **0.017** |
| Age | 0.111 | 1.188 | [1.042, 1.200] | 0.036 | 0.002 |
| BMI | 0.101 | 1.106 | [1.003, 1.220] | 0.050 | 0.042 |
| Plasma glucose | 0.111 | 1.117 | [0.745, 1.675] | 0.207 | 0.592 |
| Multiparity | 0.065 | 1.067 | [0.666, 1.709] | 0.240 | 0.787 |
| Total testosterone | 0.048 | 1.049 | [0.986, 1.116] | 0.032 | 0.128 |
| AFC | 0.000 | 1.000 | [0.957, 1.046] | 0.023 | 0.990 |
| AMH | -0.029 | 0.972 | [0.908, 1.040] | 0.035 | 0.408 |
| Total Vitamin D | -0.027 | 0.986 | [0.925, 1.024] | 0.026 | 0.297 |

**Supplementary Table 1c:** Multivariate logistic regression for binary LDL and GDM incidence (Model A)

|  | B | OR | 95% CI | Standard Error | *p*-Value |
| --- | --- | --- | --- | --- | --- |
| LDL binary | 0.594 | 1.812 | [1.196, 2.745] | 0.212 | **0.005** |
| Age | 0.115 | 1.122 | [1.056, 1.191] | 0.031 | <0.001 |
| BMI | 0.030 | 1.031 | [0.943, 1.127] | 0.045 | 0.503 |
| Plasma glucose | 0.356 | 1.427 | [0.946, 2.154] | 0.210 | 0.090 |
| LH | -0.068 | 0.934 | [0.852, 1.023] | 0.047 | 0.142 |
| E2 | -0.007 | 0.993 | [0.980, 1.004] | 0.006 | 0.234 |

**Supplementary Table 1d:** Multivariate logistic regression for binary LDL and GDM incidence (Model B)

|  | B | OR | 95% CI | Standard Error | *p*-Value |
| --- | --- | --- | --- | --- | --- |
| LDL binary | 0.710 | 2.033 | [1.255, 3.293] | 0.246 | **0.004** |
| Age | 0.110 | 1.116 | [1.040, 1.198] | 0.036 | 0.002 |
| BMI | 0.091 | 1.095 | [0.992, 1.209] | 0.050 | 0.071 |
| Plasma glucose | 0.128 | 1.136 | [0.750, 1.722] | 0.212 | 0.547 |
| Multiparity | 0.082 | 1.086 | [0.678, 1.740] | 0.241 | 0.732 |
| Total testosterone | 0.048 | 1.049 | [0.986, 1.116] | 0.032 | 0.132 |
| AFC | 0.001 | 1.001 | [0.957, 1.046] | 0.023 | 0.972 |
| AMH | -0.027 | 0.973 | [0.910, 1.041] | 0.034 | 0.434 |
| Total Vitamin D | -0.024 | 0.976 | [0.928, 1.027] | 0.026 | 0.356 |

**Supplementary Tables 2a-d: Multivariate logistic regression for HDL**

Comparisons were made using multivariate logistic regression. AFC = antral follicle count; AMH = anti-Müllarian hormone; B = Regression coefficient; BMI = body mass index; CI = Confidence Interval for OR; E2 = estradiol; GDM = gestational diabetes mellitus; HDL = high-density lipoproteins; LH = luteinizing hormone; OR = odds ratio.

**Supplementary Table 2a:** Multivariate logistic regression for continuous HDL and GDM incidence (Model A)

|  | B | OR | 95% CI | Standard Error | *p*-Value |
| --- | --- | --- | --- | --- | --- |
| HDL continuous | -0.842 | 0.431 | [0.221, 0.839] | 0.340 | **0.013** |
| Age | 0.127 | 1.135 | [1.070, 1.205] | 0.030 | <0.001 |
| BMI | 0.000 | 1.000 | [0.909, 1.100] | 0.049 | 0.994 |
| Plasma glucose | 0.339 | 1.403 | [0.940, 2.094] | 0.204 | 0.097 |
| LH | -0.059 | 0.943 | [0.861, 1.031] | 0.046 | 0.198 |
| E2 | -0.008 | 0.992 | [0.979, 1.004] | 0.006 | 0.192 |

**Supplementary Table 2b:** Multivariate logistic regression for continuous HDL and GDM incidence (Model B)

|  | B | OR | 95% CI | Standard Error | *p*-Value |
| --- | --- | --- | --- | --- | --- |
| HDL continuous | -0.991 | 0.371 | [0.168, 0.821] | 0.405 | **0.014** |
| Age | 0.112 | 1.130 | [1.054, 1.211] | 0.036 | <0.001 |
| BMI | 0.055 | 1.056 | [0.949, 1.176] | 0.055 | 0.316 |
| Plasma glucose | 0.121 | 1.128 | [0.756, 1.683] | 0.204 | 0.554 |
| Multiparity | 0.036 | 1.036 | [0.646, 1.662] | 0.241 | 0.883 |
| Total testosterone | 0.058 | 1.060 | [0.994. 1.130] | 0.033 | 0.078 |
| AFC | -0.001 | 0.999 | [0.955, 1.045] | 0.023 | 0.961 |
| AMH | -0.019 | 0.981 | [0.917, 1.050] | 0.035 | 0.580 |
| Total Vitamin D | -0.032 | 0.969 | [0.940, 1.025] | 0.026 | 0.226 |

**Supplementary Table 2c:** Multivariate logistic regression for binary HDL and GDM incidence (Model A)

|  | B | OR | 95% CI | Standard Error | *p*-Value |
| --- | --- | --- | --- | --- | --- |
| HDL binary | -0.593 | 0.553 | [0.355, 0.860] | 0.226 | **0.009** |
| Age | 0.127 | 1.135 | [1.069, 1.107] | 0.030 | <0.001 |
| BMI | 0.009 | 1.009 | [0.920, 2.056] | 0.047 | 0.846 |
| Plasma glucose | 0.317 | 1.373 | [0.917, 2.056] | 0.206 | 0.124 |
| LH | -0.053 | 0.949 | [0.868, 1.037] | 0.045 | 0.246 |
| E2 | -0.008 | 0.992 | [0.979, 1.004] | 0.006 | 0.191 |

**Supplementary Table 2d:** Multivariate logistic regression for binary HDL and GDM incidence (Model B)

|  | B | OR | 95% CI | Standard Error | *p*-Value |
| --- | --- | --- | --- | --- | --- |
| HDL binary | -0.619 | 0.539 | [0.321, 0.902] | 0.263 | **0.019** |
| Age | 0.120 | 1.127 | [1.051, 1.209] | 0.036 | <0.001 |
| BMI | 0.068 | 1.070 | [0.965, 1.188] | 0.053 | 0.200 |
| Plasma glucose | 0.099 | 1.104 | [0.737, 1.654] | 0.206 | 0.631 |
| Multiparity | 0.008 | 1.008 | [0.629, 1.616] | 0.241 | 0.973 |
| Total testosterone | 0.054 | 1.055 | [0.989, 1.125] | 0.033 | 0.102 |
| AFC | 0.001 | 1.001 | [0.957, 1.047] | 0.023 | 0.969 |
| AMH | -0.020 | 0.980 | [0.916, 1.049] | 0.035 | 0.568 |
| Total Vitamin D | -0.029 | 0.971 | [0.923, 1.022] | 0.026 | 0.258 |

**Supplementary Tables 3a-d: Multivariate logistic regression for LDL/HDL**

Comparisons were made using multivariate logistic regression. AFC = antral follicle count; AMH = anti-Müllarian hormone; B = Regression coefficient; BMI = body mass index; CI = Confidence Interval for OR; E2 = estradiol; GDM = gestational diabetes mellitus; HDL = high-density lipoproteins; LDL = low-density lipoproteins; LH = luteinizing hormone; OR = odds ratio.

**Supplementary Table 3a:** Multivariate logistic regression for continuous LDL/HDL and GDM incidence (Model A)

|  | B | OR | 95% CI | Standard Error | *p*-Value |
| --- | --- | --- | --- | --- | --- |
| LDL/HDL continuous | 0.487 | 1.628 | [1.244, 2.130] | 0.137 | **<0.001** |
| Age | 0.119 | 1.127 | [1.061, 1.196] | 0.031 | <0.001 |
| BMI | -0.004 | 0.996 | [0.907, 1.093] | 0.048 | 0.933 |
| Plasma glucose | 0.309 | 1.362 | [0.914, 2.030] | 0.204 | 0.129 |
| LH | -0.064 | 0.938 | [0.857, 1.028] | 0.046 | 0.170 |
| E2 | -0.007 | 0.993 | [0.981, 1.005] | 0.006 | 0.252 |

**Supplementary Table 3b:** Multivariate logistic regression for continuous LDL/HDL and GDM incidence (Model B)

|  | B | OR | 95% CI | Standard Error | *p*-Value |
| --- | --- | --- | --- | --- | --- |
| LDL/HDL continuous | 0.560 | 1.750 | [1.286, 2.383] | 0.157 | **<0.001** |
| Age | 0.114 | 1.121 | [1.044, 1.203] | 0.036 | 0.002 |
| BMI | 0.054 | 1.056 | [0.952, 1.171] | 0.053 | 0.305 |
| Plasma glucose | 0.081 | 1.084 | [0.724, 1.623] | 0.206 | 0.694 |
| Multiparity | 0.066 | 1.068 | [0.664, 1.718] | 0.242 | 0.786 |
| Total testosterone | 0.052 | 1.054 | [0.989, 1.123] | 0.032 | 0.106 |
| AFC | -0.002 | 0.998 | [0.953, 1.044] | 0.023 | 0.914 |
| AMH | -0.026 | 0.974 | [0.909, 1.043] | 0.035 | 0.453 |
| Total Vitamin D | -0.026 | 0.974 | [0.926, 1.025] | 0.026 | 0.320 |

**Supplementary Table 3c:** Multivariate logistic regression for binary LDL/HDL and GDM incidence (Model A)

|  | B | OR | 95% CI | Standard Error | *p*-Value |
| --- | --- | --- | --- | --- | --- |
| LDL/HDL binary | 0.671 | 1.957 | [1.258, 3.044] | 0.225 | **0.003** |
| Age | 0.124 | 1.132 | [1.067, 1.202] | 0.030 | <0.001 |
| BMI | -0.001 | 0.999 | [0.910, 1.097] | 0.048 | 0.981 |
| Plasma glucose | 0.335 | 1.398 | [0.932, 2.099] | 0.207 | 0.106 |
| LH | -0.062 | 0.940 | [0.858, 1.029] | 0.046 | 0.181 |
| E2 | -0.008 | 0.992 | [0.980, 1.004] | 0.006 | 0.215 |

**Supplementary Table 3d:** Multivariate logistic regression for binary LDL/HDL and GDM incidence (Model B)

|  | B | OR | 95% CI | Standard Error | *p*-Value |
| --- | --- | --- | --- | --- | --- |
| LDL/HDL binary | 0.782 | 2.186 | [1.308, 3.656] | 0.262 | **0.003** |
| Age | 0.122 | 1.130 | [1.053, 1.212] | 0.036 | <0.001 |
| BMI | 0.055 | 1.057 | [0.952, 1.173] | 0.053 | 0.300 |
| Plasma glucose | 0.096 | 1.100 | [0.734, 1.648] | 0.206 | 0.643 |
| Multiparity | 0.034 | 1.034 | [0.644, 1.660] | 0.241 | 0.886 |
| Total testosterone | 0.048 | 1.049 | [0.986, 1.116] | 0.032 | 0.131 |
| AFC | 0.001 | 1.001 | [0.957, 1.047] | 0.023 | 0.964 |
| AMH | -0.025 | 0.975 | [0.911, 1.044] | 0.035 | 0.476 |
| Total Vitamin D | -0.027 | 0.973 | [0.944, 1.029] | 0.026 | 0.297 |

**Supplementary Tables 4a-d: Multivariate logistic regression for TC/HDL**

Comparisons were made using multivariate logistic regression. AFC = antral follicle count; AMH = anti-Müllarian hormone; B = Regression coefficient; BMI = body mass index; CI = Confidence Interval for OR; E2 = estradiol; GDM = gestational diabetes mellitus; HDL = high-density lipoproteins; LH = luteinizing hormone; OR = odds ratio; TC = total cholesterol.

**Supplementary Table 4a:** Multivariate logistic regression for continuous TC/HDL and GDM incidence (Model A)

|  | B | OR | 95% CI | Standard Error | *p*-Value |
| --- | --- | --- | --- | --- | --- |
| TC/HDL continuous | 0.373 | 1.452 | [1.164, 1.811] | 0.113 | **<0.001** |
| Age | 0.120 | 1.127 | [1.062, 1.197] | 0.030 | <0.001 |
| BMI | -0.005 | 0.995 | [0.906, 1.093] | 0.048 | 0.925 |
| Plasma glucose | 0.319 | 1.376 | [0.925, 2.047] | 0.203 | 0.115 |
| LH | -0.066 | 0.936 | [0.854, 1.025] | 0.046 | 0.153 |
| E2 | -0.007 | 0.993 | [0.981, 1.005] | 0.006 | 0.259 |

**Supplementary Table 4b:** Multivariate logistic regression for continuous TC/HDL and GDM incidence (Model B)

|  | B | OR | 95% CI | Standard Error | *p*-Value |
| --- | --- | --- | --- | --- | --- |
| TC/HDL continuous | 0.429 | 1.536 | [1.196, 1.973] | 0.128 | **<0.001** |
| Age | 0.114 | 1.121 | [1.045, 1.203] | 0.036 | 0.001 |
| BMI | 0.053 | 1.054 | [0.950, 1.170] | 0.053 | 0.320 |
| Plasma glucose | 0.096 | 1.101 | [0.737, 1.644] | 0.205 | 0.638 |
| Multiparity | 0.053 | 1.054 | [0.655, 1.695] | 0.243 | 0.829 |
| Total testosterone | 0.053 | 1.054 | [0.990, 1.122] | 0.032 | 0.101 |
| AFC | -0.003 | 0.997 | [0.953, 1.044] | 0.023 | 0.909 |
| AMH | -0.025 | 0.975 | [0.910, 1.004] | 0.035 | 0.468 |
| Total Vitamin D | -0.025 | 0.976 | [0.927, 1.027] | 0.026 | 0.343 |

**Supplementary Table 4c:** Multivariate logistic regression for binary TC/HDL and GDM incidence (Model A)

|  | B | OR | 95% CI | Standard Error | *p*-Value |
| --- | --- | --- | --- | --- | --- |
| TC/HDL binary | 0.664 | 1.942 | [1.243, 3.034] | 0.228 | **0.004** |
| Age | 0.122 | 1.130 | [1.064, 1.199] | 0.030 | <0.001 |
| BMI | -0.001 | 0.999 | [0.910, 1.097] | 0.048 | 0.984 |
| Plasma glucose | 0.311 | 1.364 | [0.912, 2.042] | 0.206 | 0.131 |
| LH | -0.067 | 0.935 | [0.853, 1.025] | 0.047 | 0.150 |
| E2 | -0.008 | 0.992 | [0.980, 1.005] | 0.006 | 0.212 |

**Supplementary Table 4d:** Multivariate logistic regression for binary TC/HDL and GDM incidence (Model B)

|  | B | OR | 95% CI | Standard Error | *p*-Value |
| --- | --- | --- | --- | --- | --- |
| TC/HDL binary | 0.817 | 2.264 | [1.346, 3.807] | 0.265 | **0.002** |
| Age | 0.118 | 1.125 | [1.049, 1.207] | 0.036 | 0.001 |
| BMI | 0.055 | 1.056 | [0.952, 1.172] | 0.053 | 0.302 |
| Plasma glucose | 0.076 | 1.079 | [0.721, 1.615] | 0.206 | 0.712 |
| Multiparity | 0.046 | 1.047 | [0.652, 1.681] | 0.242 | 0.850 |
| Total testosterone | 0.053 | 1.054 | [0.988, 1.125] | 0.033 | 0.110 |
| AFC | 0.001 | 1.001 | [0.957, 1.047] | 0.023 | 0.953 |
| AMH | -0.031 | 0.969 | [0.905, 1.038] | 0.035 | 0.372 |
| Total Vitamin D | -0.027 | 0.973 | [0.925, 1.024] | 0.026 | 0.292 |

**Supplementary Tables 5a-d: Multivariate logistic regression for TG/HDL**

Comparisons were made using multivariate logistic regression. AFC = antral follicle count; AMH = anti-Müllarian hormone; B = Regression coefficient; BMI = body mass index; CI = Confidence Interval for OR; E2 = estradiol; GDM = gestational diabetes mellitus; HDL = high-density lipoproteins; LH = luteinizing hormone; OR = odds ratio; TG = triglycerides.

**Supplementary Table 5a:** Multivariate logistic regression for continuous TG/HDL and GDM incidence (Model A)

|  | B | OR | 95% CI | Standard Error | *p*-Value |
| --- | --- | --- | --- | --- | --- |
| TG/HDL continuous | 0.119 | 1.126 | [0.919, 1.381] | 0.104 | **0.253** |
| Age | 0.121 | 1.128 | [1.063, 1.197] | 0.030 | <0.001 |
| BMI | 0.034 | 1.034 | [0.945, 1.133] | 0.046 | 0.466 |
| Plasma glucose | 0.370 | 1.448 | [0.968, 2.165] | 0.205 | 0.071 |
| LH | -0.066 | 0.936 | [0.855, 1.025] | 0.046 | 0.155 |
| E2 | -0.008 | 0.992 | [0.979, 1.004] | 0.006 | 0.192 |

**Supplementary Table 5b:** Multivariate logistic regression for continuous TG/HDL and GDM incidence (Model B)

|  | B | OR | 95% CI | Standard Error | *p*-Value |
| --- | --- | --- | --- | --- | --- |
| TG/HDL continuous | 0.148 | 1.160 | [0.927, 1.451] | 0.114 | **0.194** |
| Age | 0.115 | 1.122 | [1.047, 1.203] | 0.035 | 0.001 |
| BMI | 0.095 | 1.100 | [0.995, 1.216] | 0.051 | 0.062 |
| Plasma glucose | 0.146 | 1.157 | [0.776, 1.725] | 0.204 | 0.474 |
| Multiparity | 0.034 | 1.035 | [0.646, 1.656] | 0.240 | 0.887 |
| Total testosterone | 0.053 | 1.054 | [0.991, 1.121] | 0.031 | 0.094 |
| AFC | -0.001 | 0.999 | [0.955, 1.045] | 0.023 | 0.961 |
| AMH | -0.022 | 0.979 | [0.914, 1.023] | 0.035 | 0.532 |
| Total Vitamin D | -0.028 | 0.972 | [0.943, 1.028] | 0.026 | 0.276 |

**Supplementary Table 5c:** Multivariate logistic regression for binary TG/HDL and GDM incidence (Model A)

|  | B | OR | 95% CI | Standard Error | *p*-Value |
| --- | --- | --- | --- | --- | --- |
| TG/HDL binary | 0.537 | 1.710 | [1.104, 2.649] | 0.223 | **0.016** |
| Age | 0.120 | 1.127 | [1.062, 1.196] | 0.030 | <0.001 |
| BMI | 0.013 | 1.013 | [0.924, 1.110] | 0.047 | 0.788 |
| Plasma glucose | 0.372 | 1.451 | [0.980, 2.150] | 0.200 | 0.063 |
| LH | -0.066 | 0.936 | [0.855, 1.024] | 0.046 | 0.149 |
| E2 | -0.008 | 0.993 | [0.979, 1.004] | 0.006 | 0.201 |

**Supplementary Table 5d:** Multivariate logistic regression for binary TG/HDL and GDM incidence (Model B)

|  | B | OR | 95% CI | Standard Error | *p*-Value |
| --- | --- | --- | --- | --- | --- |
| TG/HDL binary | 0.731 | 2.076 | [1.240, 3.475] | 0.263 | **0.005** |
| Age | 0.116 | 1.123 | [1.048, 1.205] | 0.036 | 0.001 |
| BMI | 0.063 | 1.066 | [0.962, 1.180] | 0.052 | 0.221 |
| Plasma glucose | 0.168 | 1.183 | [0.797, 1.757] | 0.202 | 0.405 |
| Multiparity | 0.070 | 1.072 | [0.669, 1.720] | 0.241 | 0.772 |
| Total testosterone | 0.056 | 1.057 | [0.991, 1.128] | 0.033 | 0.092 |
| AFC | 0.000 | 1.000 | [0.955, 1.047] | 0.023 | 0.992 |
| AMH | -0.026 | 0.974 | [0.910, 1.043] | 0.035 | 0.456 |
| Total Vitamin D | -0.024 | 0.975 | [0.928, 1.027] | 0.026 | 0.358 |
