# Supplementary material for: Pre-pregnancy LDL/HDL and total Cholesterol/HDL ratios are strong predictors of gestational diabetes mellitus in women undergoing assisted reproductive technologies
Source: Reprod Biol Endocrinol. 2024 Dec 5;22:155. doi: 10.1186/s12958-024-01320-9 (PMC11619337; doi:10.1186/s12958-024-01320-9)
Supplement: Supplementary file 2 — Supplementary Material 2 [file 12958_2024_1320_MOESM2_ESM.pdf]

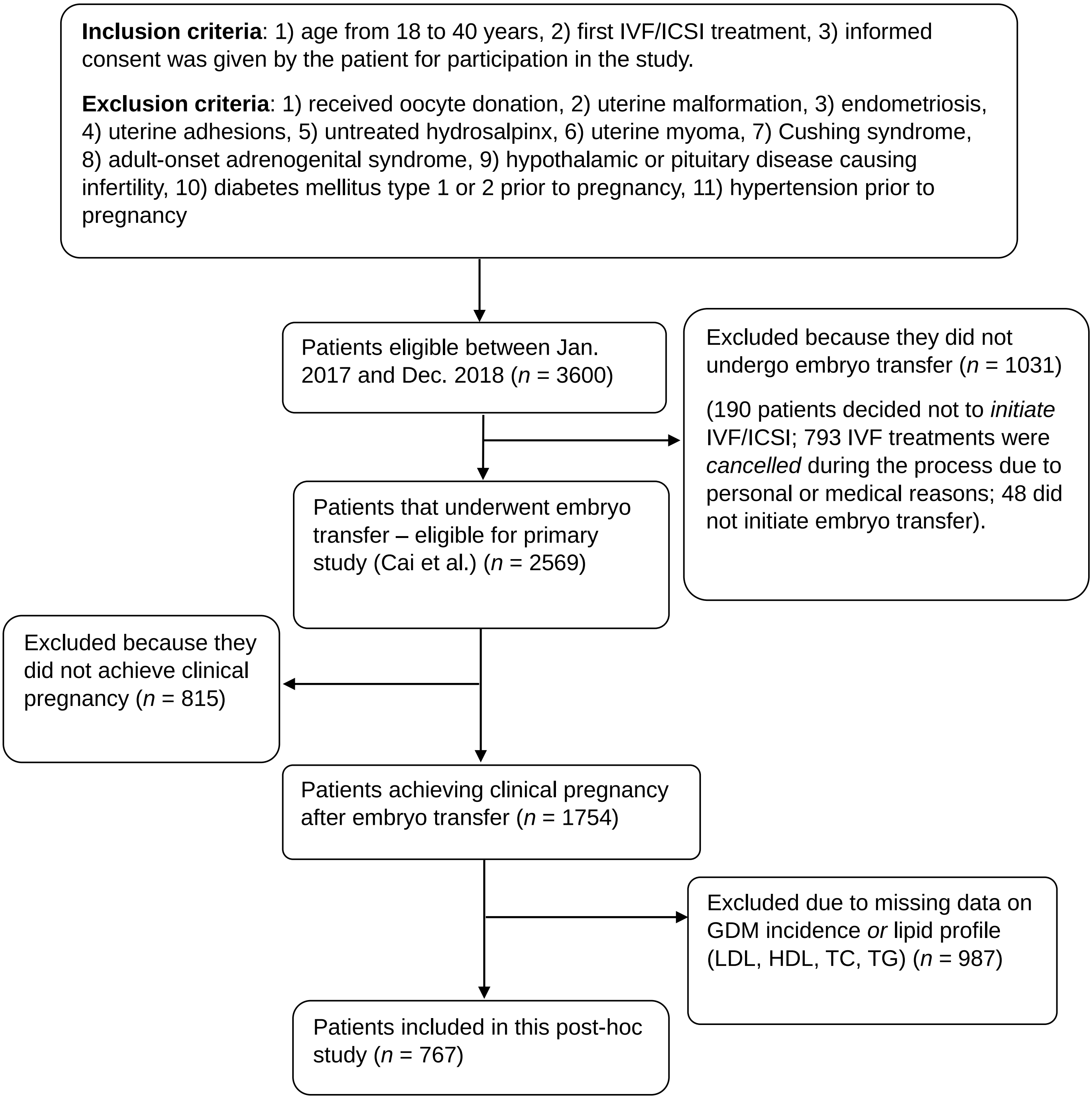

Supplementary Figure 1: Study flowchart with inclusion and exclusion criteria

Abbreviations: GDM = gestational diabetes mellitus; ICSI = intracytoplasmic sperm injection; IVF = in-vitro fertilization; LDL = low-density lipoproteins; HDL = high-density lipoproteins; TC = total cholesterol; TG = triglycerides.

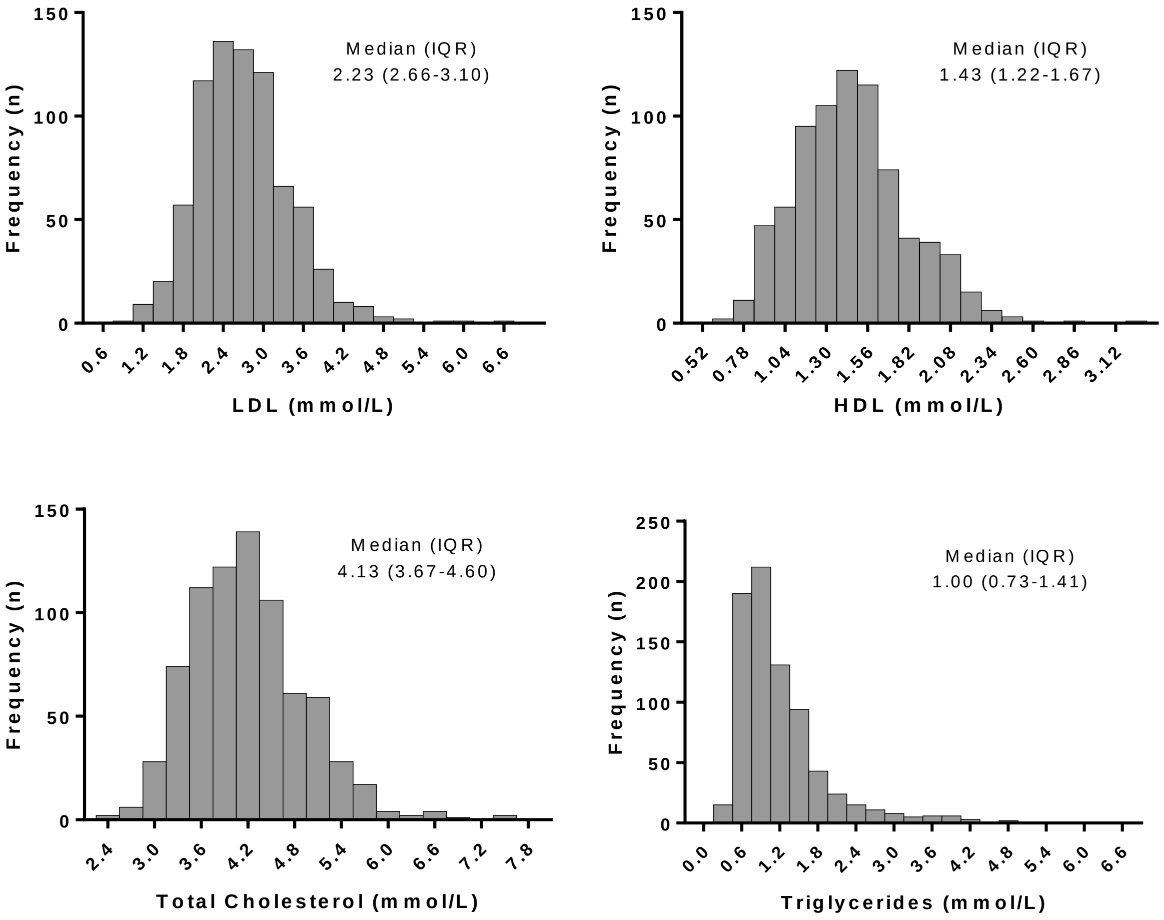

Supplementary Figures 2a-d: Distribution of lipids in the study population

Data is presented as a histogram with frequency, *n* (%), and median with IQR. Abbreviations: IQR = interquartile range; LDL = low-density lipoproteins; HDL = high-density lipoproteins.

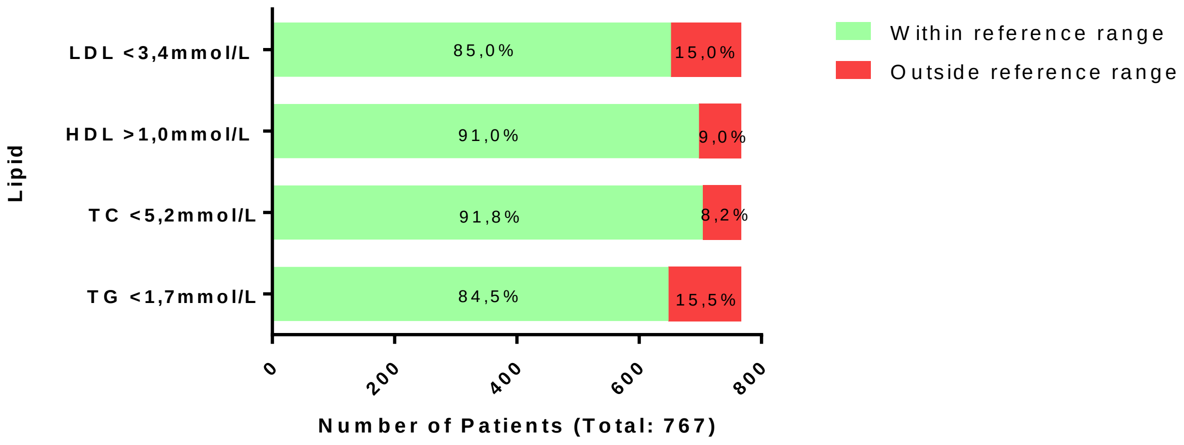

Supplementary Figure 3: Distribution of lipids according to guidelines

Lipid values were evaluated according to the AACE (American Association of Clinical Endocrinologists and American College of Endocrinology) 2017 guidelines and the 2016 Chinese guidelines for the management of dyslipidemia in adults. Data is presented as frequency, *n* (%). Abbreviations: LDL = low-density lipoproteins; HDL = high-density lipoproteins; TC = total cholesterol; TG = triglycerides.

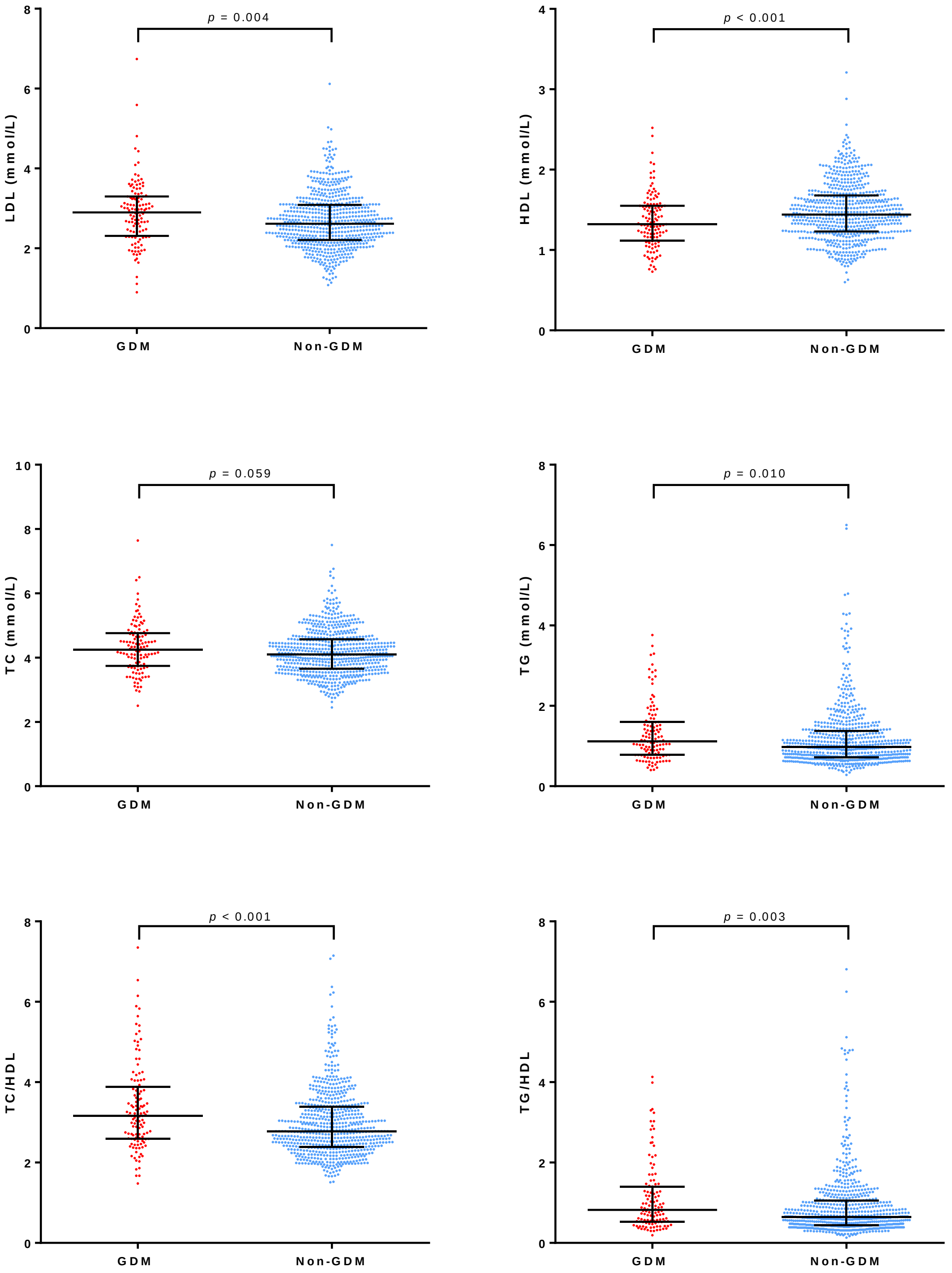

Supplementary Figures 4a-f: Lipid parameters in GDM versus non-GDM patients

Plots are presented as median and IQR (interquartile range). *p*-values were calculated with the Mann-Whitney U test. GDM = gestational diabetes mellitus; HDL = high-density lipoproteins; LDL = low-density lipoproteins; TC = total cholesterol; TG = triglycerides.

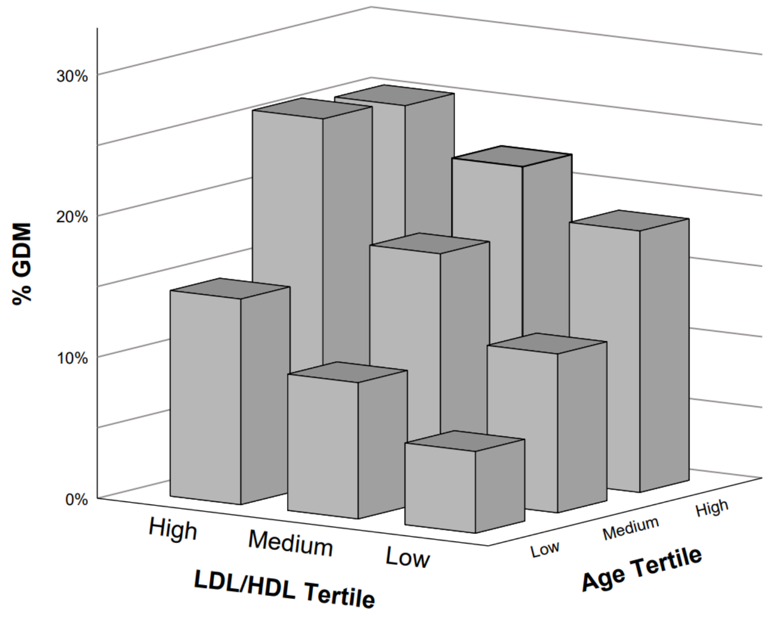

30%
20%
10%
0%
% GDM
High
Medium
Low
LDL/HDL Tertile
Low
Medium
High
Age Tertile

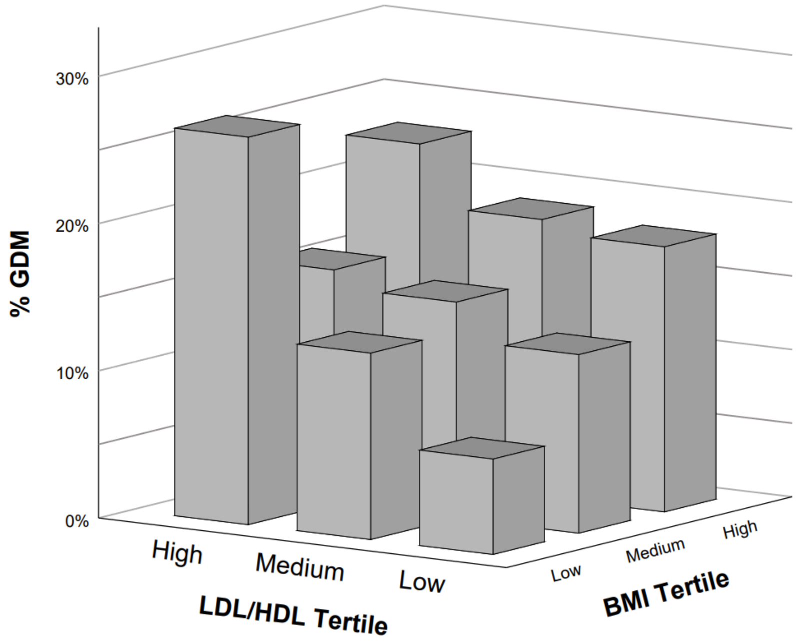

30%
20%
10%
0%
% GDM
High
Medium
Low
LDL/HDL Tertile
Low
Medium
High
BMI Tertile

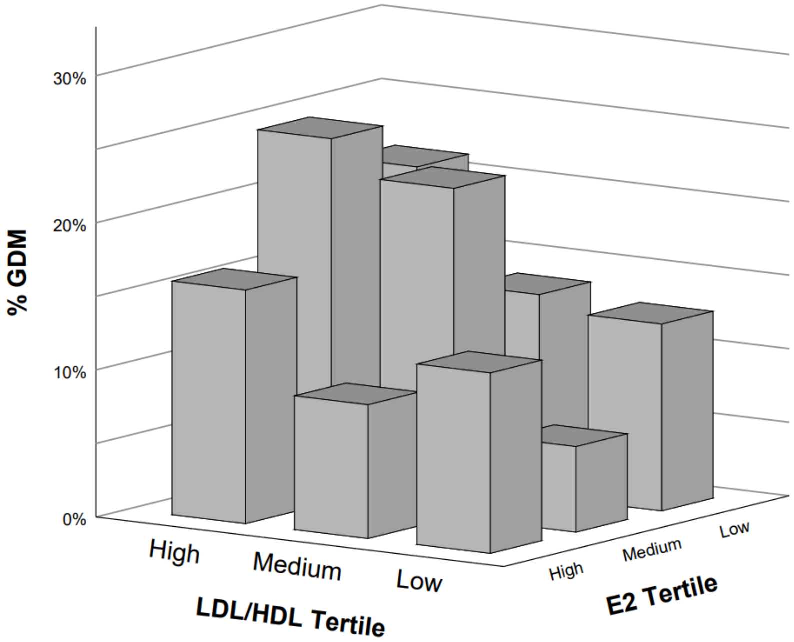

Supplementary Figures 5a-c: Effects of LDL/HDL ratio and other GDM risk factors (age, BMI, and E2) on GDM incidence in women undergoing IVF/ICSI

Abbreviations: BMI = body mass index; GDM = gestational diabetes mellitus; E2 = estradiol; HDL = high-density lipoproteins; ICSI = intracytoplasmic sperm injection; IVF = in-vitro fertilization; LDL = low-density lipoproteins.
